# Performance of steroid eluting bipolar epicardial leads in pediatric and congenital heart disease patients: 15 years of single center experience

**DOI:** 10.1186/1749-8090-9-84

**Published:** 2014-05-12

**Authors:** Christian Paech, Martin Kostelka, Ingo Dähnert, Patrick Flosdorff, Frank Thomas Riede, Roman Antonin Gebauer

**Affiliations:** 1 Department for Pediatric Cardiology, University of Leipzig - Heart Center, Strümpellstr. 39, 04289 Leipzig, Germany; 2 Department for Cardiac Surgery, University of Leipzig - Heart Center, Leipzig, Germany

**Keywords:** Epicardial leads, Pacing, Congenital heart disease, Pediatrics

## Abstract

**Objective:**

Cardiac pacing is sometimes required for patients with congenital heart disease for various reasons. Because of complex anatomy, repetitive previous heart surgery and patient size, epicardial leads are of special importance in these patients. Using epicardial leads has been discussed controversly regarding pacing performance and lead survival. The aim of this study was to review the long-term data on pacing performance as well as lead survival of epicardial leads implanted in our center.

**Methods:**

Retrospective chart review and review of the literature.

**Results:**

82 consecutive pediatric patients or adult patients with congenital heart disease with 158 epicardial leads (Medtronic 4968, bipolar, steroid - eluting) were included. We found 1.2% pacemaker-related early postoperative complications. The incidence of lead dysfunction was 7.5% (12/158) for primary (i.e. directly related to the lead itself) lead dysfunction and 3.2% (5/158) of lead abandonment for reasons not directly related to the lead itself. Primary lead dysfunction occured after a median of 3.83 years. Lead survival at 2, 5 and 10 years was 98.7%, 93% and 92.4%. There were no infections reported. Stable median measurements for impedance (RA/RV/LV of 577/483/610 Ohm), sensing threshold (RA/RV/LV of 2.0/11.0/10.0 mV) and pacing threshold (RA/RV/LV of 0.75 V at 0.4 ms/1.0 V at 0.49 ms/1.0 V at 0.45 ms) indicated a good mid- to longterm performance. The only risk factor for primary lead dysfunction was young age at implantation.

**Conclusion:**

The use of epicardial leads in pediatric and adult patients with congenital heart disease shows good longterm outcomes in terms of pacing performance and lead survival. The authors encourage using epicardial leads in patients with congenital heart disease based on the patient‘s individual characteristics.

## Background

Cardiac pacing is required rather frequently in patients with congenital heart disease (CHD) for various reasons, ranging from symptomatic bradycardia caused by sinus node dysfunction over postoperative complete heart block to cardiac resynchronization therapy. Although transvenous implantation of a cardiac pacemaker is technically feasible and less invasive than surgical placement of epicardial leads, there are several advantages of epicardial leads in pediatric patients or older patients with CHD. Most congenital heart disease patients will need cardiac pacing throughout their whole life. In view of the limited lead survival both in epicardial and endocardial leads, as well as the necessary relocation of the leads due to the natural growth of children, these patients expect lead removal and reimplantation several times in their lives [1]. Some of these patients require repetitive surgical procedures and cardiac catheterizations for other reasons than pacemaker implantation. It seems of utmost importance to save venous access taking into account hemodynamics and future catheter or transvenous pacing lead placement in those patients. In addition, it is relatively easy and only associated with a low risk to place epicardial leads during cardiac surgery of the underlying congenital heart disease. As recent data support, epicardial pacing provides the possibility for left apical pacing. Left apical pacing is reported as the superior pacing site in view of pacing induced dyssynchrony [2]. In infants and neonates epicardial lead placement leaves the possibility for abdominal device implantation which provides enough room to host sufficient wire reserve to match the child’s growth and serves as bridge to a transvenous pacemaker system at an older age, when the risk for venous occlusion, thrombosis or impairment of venous flow is lower.

In this cohort the patients can be assigned to one of four main groups requiring pacemaker implantation. The first group consists of children with congenital heart disease after surgical procedures, for either repair or palliation with surgical induced complete heart block. The second group includes patients who suffer from congenital anatomic cardiac malformation like corrected congenital transposition of the great arteries, heterotaxia or atrial isomerism resulting in sinus node dysfunction or complete heart block. The third group contains those patients with intraventricular or interventricular dyssynchrony who require pacing for cardiac resynchronization, whereas the fourth group includes patients without structural heart disease and complete heart block or asystole during breath-holding spells.

The aim of this study was to evaluate the mid- to longterm lead survival and performance in these special groups of patients and to review the literature for current strategies of epicardial versus endocardial lead placement.

## Methods

### Data collection

The data of 82 consecutive patients who were either implanted or looked after at the division of pediatric cardiology, University of Leipzig, Heart Center, were evaluated retrospectively. Included were all patients who received a permanent pacemaker system with epicardial leads, either atrial or ventricular from 1996 to 2010. Inclusion required at least one structured follow-up performed for at least one time after implantation and at least 1 month after implantation. Follow-up contained a 12-lead ECG, a device interrogation with measurement of thresholds, sensing and lead impedance, chest x-ray, physical examination and the patient’s history. Patients with an ICD and patients with unipolar pacemaker leads (n = 2) were excluded from this study. The collected data were reviewed by two experienced pediatric cardiologists, one of them specialized in pediatric heart rhythm and device therapy. The review included the surgical report and all available follow-up data. Whenever a patient received follow-up at another center, the structured follow-up concerning lead data ended. However, further information concerning lead complications was obtained from those patients.

### Definitions

Lead dysfunction was differentiated in primary dysfunction and lead abandonment. Primary lead dysfunction included problems directly related to the lead itself such as exit block due to lead fracture, isolation defects or dislocation. Lead abandonment included reasons not directly related to the lead itself such as infection, elective replacement for other reasons or dislocation by an external trauma.

### Statistics

Data analysis was performed using IBM SPSS 20.0 Software. Patients were compared using Student *t*-test or Mann–Whitney test for continuous variables and chi-square test or Fisher’s exact test for binary variables. The Fisher exact test was utilized in place of chi-square test when the expected frequency of a cell within a contingency table was <5. Kaplan – Meier plots were used to illustrate lead survival. A p-value of < 0.05 was considered statistically significant. All electrode measurements are depicted as median with standard deviation. The survival data are depicted as median with range.

## Results

### Patients characteristics

82 patients meeting all inclusion and no exclusion criteria were identified. This accounted for 158 epicardial pacemaker leads (Medtronic 4968, bipolar, steroid - eluting n = 158). All leads were connected to a standard cardiac pacemaker device of one of the following companies: Medtronic Inc., Biotronik or St. Jude Medical. All patients were treated perioperatively with cephazoline (50 mg/kg every 8 h) for at least 24 h, mostly until central venous lines were removed.

61 patients suffered from congenital heart disease, while 21 patients showed anatomically normal hearts. There were 42 female and 40 male patients with a median age of 7.64 years (range 1.08 – 54.7) and a median age at primary implantation of a cardiac pacemaker device of 1.95 years (range 0.01 – 48.5). Median time of follow-up was 3.3 years (range 0.1 – 15.2).

### Indication

Indications for lead implantation were postoperative complete heart block in 33 patients, sick sinus syndrome in 19 patients, congenital complete heart block in 14 patients, complete heart block in association with corrected congenital transposition of the great arteries in 9 patients, breath-holding spells in 5 patients and 2 patients received lead implantation during the course of a cardiac resynchronization therapy. Indications are depicted in Table 1.

**Table 1:** Indication groups for cardiac pacing

**Indication groups**	**Diagnosis or surgical procedure**	**Number of patients per indication group (% of all 82 patients)**
Congenital heart block		**23 (28)**
	*normal cardiac anatomy*	14 (17)
	*ccTGA*	9 (11)
Postoperative heart block		**33 (40)**
	*Ventricular septal defect*	*10 (12)*
	*Complete atrico ventricular septal defect*	*5 (6)*
	*Subaortic stenosis*	*1 (1)*
	*Arterial switch*	*5 (6)*
	*Tetralogy of Fallot*	*1 (1)*
	*others*	*11 (14)*
Sick sinus syndrome		**19 (24)**
	*uncorrected CHD*	*3 (4)*
	*Heterotaxia*	*2 (3)*
	*post- Fontan or TCPC Procedure*	*5 (6)*
	*others*	*9 (11)*
Resynchronization therapy		**2 (2)**
	*Pacing induced*	*1 (1)*
	*End stage CHD*	*1 (1)*
Breath holding spells		**5 (6)**

The left column shows the indication groups for cardiac pacing. The middle column shows the surgical procedures or main congenital anomalies of each indication group. The right column shows the number of patients per indication group (bold figures) and number of patients per surgical procedure or main congenital anomaly within each indication group.

### Mortality rate

There was no death related to a dysfunction of either the cardiac pacemaker device or a lead dysfunction.

### Early complications

Reviewing all 82 patients, only one (1.2%) pacemaker-related postoperative complication was reported. The affected patient had to undergo surgical revision due to a pocket hematoma.

### Late complications

Two cases of coronary compression during follow-up occured. Both patients required re-operation with repositioning of the epicardial pacing leads. The first patient was a female infant with congenital complete heart block caused by maternal lupus erythematodes who underwent implantation of epicardial VVI pacemaker at the age of 3 months and received an additional atrial epicardial electrode at the age of 1.75 years. Four months later, she developed dilative cardiomyopathy and was listed for cardiac transplantation. Selective angiography of the left coronary artery revealed cardiac strangulation caused by the right ventricular electrode. Compression of the left anterior descending artery and circumflex artery was relieved by repositioning the right ventricular electrode. A left ventricular electrode and a biventricular pacemaker were inserted for resynchronization therapy. On discharge, the interventricular synchrony was improved. Despite the intial improvement she developed spastic tetraparesis one month after dismissal and died 9 month later due to non-cardiac reasons.

**Figure 1:**
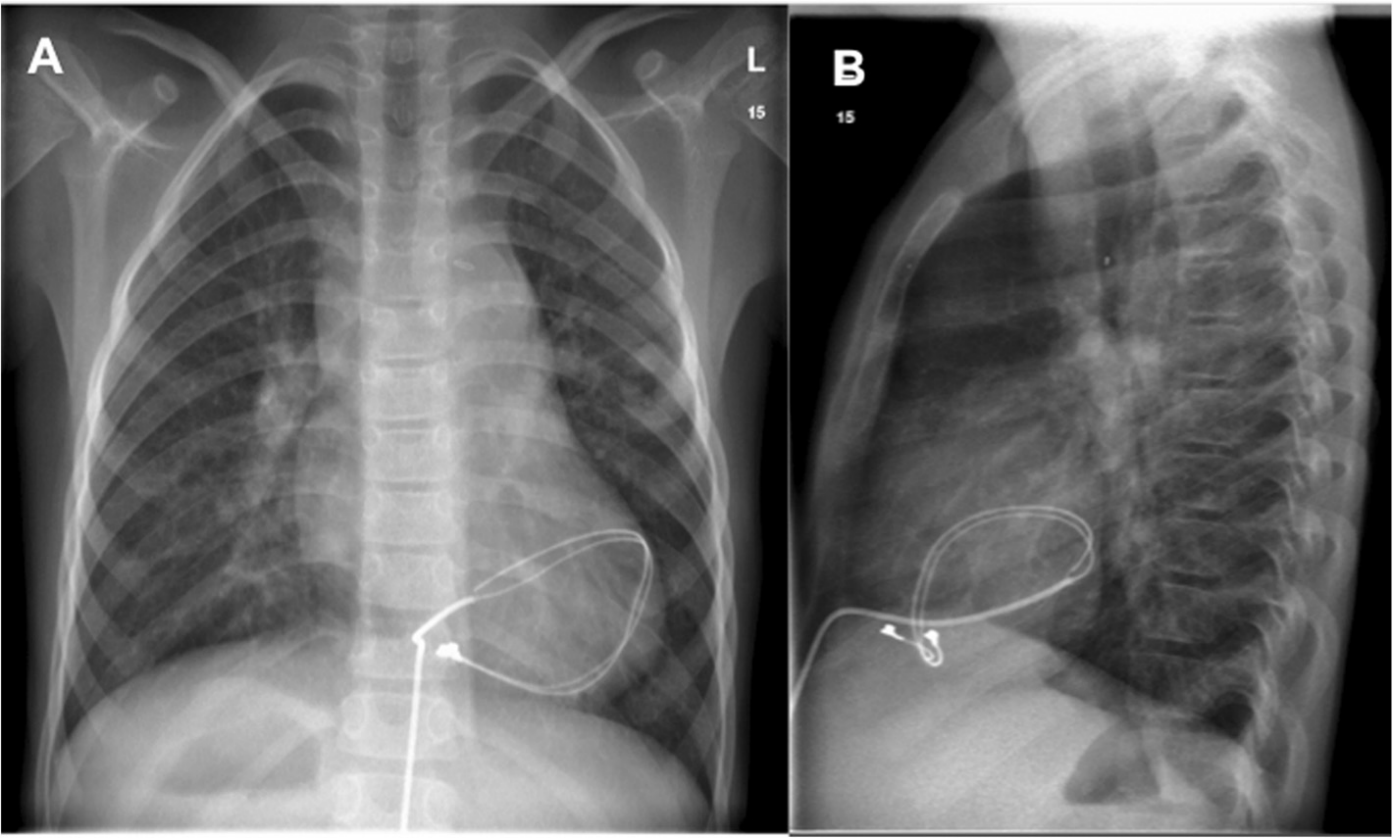
**Shows the chest x-ray of the second patient with cardiac strangulation by an epicardial pacemaker lead.** (For details read Results). **A**: Chest x-ray a.p. projection. **B**: Lateral projection showing the abnormal course of the RV lead, which forms a loop around the left ventricle. Note the course of the lead within the border of the heart shadow in panel **B**.

The second patient was an 8-year-old boy with complex congenital malformation, including a left atrial isomerism, polysplenia, sick sinus syndrome and anomalous venous drainage via a prominent vena azygos. Intracardiac lesions consisted of a ventricular septal defect, a common atrium and a non- compaction of the apical left ventricle. Pacemaker implantation had been performed at the age of 5 days. Chest x-ray showed an anomalous course of the epicardial RV pacemaker lead forming a loop around the left ventricle causing a diastolic LV constriction (Figure 1). Cardiac catheterization showed compression of the circumflex artery. In the absence of impaired left ventricular wall motion, negative troponine and no signs of myocardial ischemia, the pacemaker lead was exchanged for a new epicardial two chamber device. The old lead was cut off. The patient did well during follow up. Both patients had received primary pacemaker implantation at another hospital. In summary, cardiac strangulation is a rare complication of epicardial pacing [3].

### Lead failure

**Figure 2:**
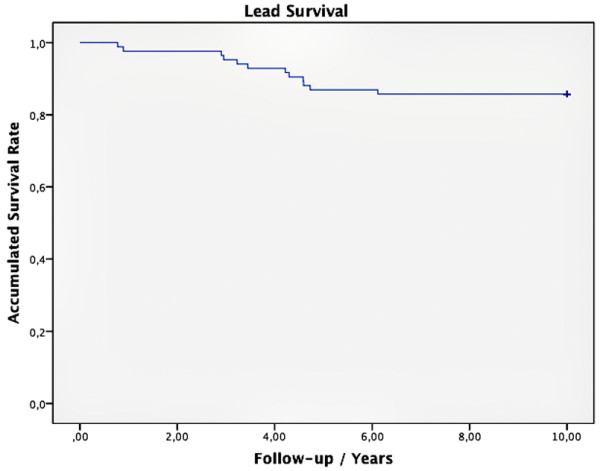
**Depicts a Kaplan-Meier curve of lead survival.** Starting with 158 pacemaker leads, every step down of the curve marks a case of primary lead dysfunction or lead abandonment.

The incidence of primary lead dysfunction was 7.5% (12/158). The incidence of lead abandonment was 3.2% (5/158). The reason for primary lead dysfunction was always lead fracture (n = 12). No case of isolation defect or macrodislocation was reported. Lead abandonment was always due to lead removal for non-pathologic circumstances such as reoperation of the underlying congenital heart disease (n = 5). It is of some importance that no infections were reported in this study group. Lead survival at 2, 5 and 10 years was 98.7%, 93% and 92.4%. Primary lead dysfunction occured after a median of 3.83 years (range 0.78 – 6.12) (Figure 2). Primary lead dysfunction was not correlated to any of the following variables; (1) sex (p = 0.12); (2) structural normal heart versus congenital heart disease (p = 0.49); (3) lead position seperated into right ventricular apex, right ventricular free wall, right ventricular outflow tract, left ventricular apex or left ventricular free wall (right ventricular positions p = 0.62, left ventricular positions p = 1.0); (4) or to the presence of a biventricular pacemaker system (p = 0.15). However, primary lead dysfunction was significantly more frequent in patients who had received implantation at younger age (p = 0.01).

### Thresholds and impedance

Pacing thresholds, sensing and impedance measurements at initial interrogation following implantation and at the patients last follow-up after a mean of 3.3 years (range 0.1 – 15.2) are depicted in Table 2.

**Table 2:** Lead data at primary implantation and follow-up

	**Pacing PI**	**Sensing PI**	**Impedance PI**	**Pacing FUP**	**Sensing FUP**	**Impedance FUP**
RA/LA	0,9 V (0,47 – 1,67), at 0,4 ms (0,31 – 0,5)	2,8 V (0,81 – 4,8)	593 Ω (410 – 776)	0,75 V (0,12 – 1,38), at 0,4 ms (0,28 – 0,52)	2,0 V (0,3 – 8,0)	577 Ω (274 – 1256)
RV	0,9 V (0,48 – 1,32), at 0,49 ms (0,34 – 0,64)	9,2 V (3,0 – 15,4)	594 Ω (245 – 913)	1,0 V (0,38 – 4,2), at 0,49 ms (0,27 – 0,7)	11,0 V (6,16 – 15,8)	482 Ω (273 – 886)
LV	0,75 V (0,25 – 1,48), at 0,5 ms (0,36 – 0,64)	9,5 V (6,6 – 18)	717 Ω (311 – 1123)	1,0 V (0,27 – 1,73), at 0,45 ms (0,24 – 0,66)	10,0 V (4,73 – 15,27)	610 Ω (463 – 757)

Table 2: RA = right atrium, LA = left atrium, RV = right ventricle, LV = left ventricle, PI = primary implantation, FUP = last follow-up.

## Discussion

This study reports on the single center experience with 158 Medtronic 4968, bipolar, steroid - eluting epicardial cardiac pacemaker leads in 82 pediatric and adult patients with CHD.

Previous studies, report an overall 2 year cumulative survival of 93% to 99% and 5 year survival of 58% to 89% respectively for epicardial leads. Lead failure in epicardial leads is reported from 8% to 36% with a wide range between centers [4-7]. As stated by Post et al., who reviewed data of 93 patients with congenital heart disease in a single center focussing on possible risk factors for lead failure, only young age at implantation proved to be a risk factor for lead dysfunction [8]. Interestingly, Murayma et al. found congenital heart disease itself to be the only predictor of lead failure in their collective of 109 pediatric patients [9].

Two single center studies investigating advantages of endocardial over epicardial leads, were carried out. Odim et al. found 18% versus 10% of lead dysfunction comparing epicardial to endocardial leads in 148 pediatric patients, whereas Fortescue et al. found 9% versus 7% of lead dysfunction comparing epicardial to endocardial leads [10,11]. Fortescue et al. also found that acute dislodgements, fractures and insulation breaks were more common in the transvenous leads group and high thresholds, chronic dislodgements and infections were more common in the epicardial leads group. In contrast, Odim et al. reported no significant differences in performance and lead survival between these groups.

By taking a closer look at the currently available data on the Medtronic 4968 epicardial steroid-eluting lead, Tomaske et al. reported the largest pediatric series with 114 children with 239 atrial and ventricular bipolar epicardial leads (Medtronic CapSure 10366 or 4968, Minneapolis, MN) enrolled. After a follow-up of up to 12.2 years (median, 3.2 years) they found low median pacing thresholds below 1.2 V at 0.5 ms and a lead survival of 85% at 5 years for atrial and 94% for ventricular leads respectively. They concluded that bipolar steroid-eluting leads provide an alternative approach for permanent pacing and may also be considered for left atrial and ventricular pacing, resynchronization, or defibrillator therapy.

In summary, the results in the presented study are consistent with the findings in previous studies. Good mid- to even longterm survival of epicardial leads as well as stable pacing thresholds, lead impedance and sensing capacity were ascertained. In comparison to other study groups, this study reported a rather low incidence of lead dysfunction of 7.5%. Reasons for this may be based on the implanting cardiac surgeon’s individual experience as well as the exclusive use of bipolar steroid eluting leads in contrast to unipolar or non drug eluting pacing leads. The only risk factor for primary lead dysfunction in this study group proved to be young age at implantation. Taking a closer look at the affected patients, nearly all of them were between 3 and 7 years of age at the time of lead dysfunction. Patients of this particular age group typically show a high degree of natural growth and physical activity. It may be speculated that this natural behavior, possibly causing many incidental injuries, might influence lead survival more than in older or much younger patients. It has to be mentioned that we found no patient at all with a device infection in the presented study group.

In conclusion, most studies conducted on this topic found that modern, i.e. steroid-eluting bipolar epicardial pacemaker leads show good long term performance and durability [12,13]. The currently available data concerning advantages and disadvantages of endocardial versus epicardial pacemaker leads remain contradicting. As the only concurrent finding, both single center comparisons mentioned above, found a lower rate of lead failure in the endocardial leads group. However, in both studies patients who received epicardial lead implantation were younger, more often affected by congenital heart disease and showed a higher rate of concomittant surgery, which might have caused the negative results in the epicardial leads group. In addition, referring to the data presented in the actual study, we report an equally low rate of lead dysfunction as Fortescue et al. and an even lower rate than Odim et al..

As there currently available data imply an equal performance, the decision on whether to implant either endo- or epicardial leads should rather be made on the basis of the patient’s individual characteristics than on technical aspects such as lead performance or durability. Moreover, preservation of vascular access, expected operations or reoperations and the spacial considerations for leaving a pacing lead reserve to compensate the patient’s growth should be particularly taken into account when choosing an acceptable route for pacemaker implantation or replacement in pediatric patients or patients with CHD [14]. In the authors’ opinion, current indications for epicardial leads are contraindications for transvenous lead placement such as limited venous access, repetitive infections of a transvenous system, small body size, intracardiac shunt or practial reasons for example expected cardiac surgery of the underlying congenital heart disease. Lately, favourable results in terms of preservation of ventricular function were reported for epicardial pacing from the left ventricular apex [15]. In all other patients, the less risky transvenous implantation route should be preferred.

## Conclusion

The use of bipolar steroid- eluting epicardial leads in the setting of pediatric and adult patients with congenital heart disease shows good longterm outcomes as far as pacing performance and lead survival are concerned. In addition, venous access is preserved for future interventions and the risk for thrombosis and infection is lower. The authors encourage using epicardial leads in patients with congenital heart disease and other pediatric patients based on the patient’s individual characteristics.

### Limitations

Three limitations to this study have to be mentioned. First of all, this is a retrospective study. Secondly, the presented data are a single center experience which might be biased due to surgical technique and internal processes. The third limitiation is due to the fact that some patients are refferenced to our center exclusively for pacemaker implantation and will be looked after in a private practice. Those patients will be presented to our centre again only in case of a complication. Although those patients are surely without pacemaker related complications, there is no structured follow-up data available.

## Consent

Written informed consent was obtained from the patient for publication of both case reports and accompanying images.

## Abbreviations

CHD: Congenital heart disease; ICD: Implantable cardioverter defibrillator; ECG: Electrocardiogram.

## Competing interests

The authors declare that they have no competing interests.

## Authors’ contributions

CP conceptualized and designed the study. MK performed a critical revision and approval of the article. ID approved the article. PF helped in the draft of the manuscript. FR did a critical revision of the atricle. RG performed the data analysis. All authors read and approved the final manuscript.

## References

[B1] Zartner PA Wiebe W Toussaint-Goetz N Schneider MB Lead removal in young patients in view of lifelong pacing Europace 2010 9 714 718 10.1093/europace/euq059 20219754

[B2] Janousek J van Geldorp IE Krupicková S Rosenthal E Nugent K Tomaske M Früh A Elders J Hiippala A Kerst G Gebauer RA Kubus P Frias P Gabbarini F Clur SA Nagel B Ganame J Papagiannis J Marek J Tisma-Dupanovic S Tsao S Nürnberg JH Wren C Friedberg M de Guillebon M Volaufova J Prinzen FW Delhaas T Permanent cardiac pacing in children: choosing the optimal pacing site: a multicenter study. Working Group for Cardiac Dysrhythmias and Electrophysiology of the Association for European Pediatric Cardiology Circulation 2013 9 5 613 623 10.1161/CIRCULATIONAHA.112.115428 23275383

[B3] Riede FT Kostelka M Dähnert I Cardiac strangulation: a rare, but devastating complication of epicardial pacing causing progressive myocardial ischaemia Eur Heart J 2009 9 4 435 18930912 10.1093/eurheartj/ehn449

[B4] Tomaske M Gerritse B Kretzers L Pretre R Dodge-Kathami A Rahn M Bauersfeld U A 12-year experience of bipolar steroid-eluting epicardial pacing leads in children Ann Thorac Surg 2008 9 5 1704 1711 10.1016/j.athoracsur.2008.02.016 18442570

[B5] Walker F Siu SC Woods S Cameron DA Webb GD HARRIS L Long-term outcomes of cardiac pacing in adults with congenital heart disease J Am Coll Cardiol 2004 9 10 1894 1901 10.1016/j.jacc.2003.12.044 15145118

[B6] Lichtenstein BJ Bichell DP Connolly DM Lamberti JJ Shepard SM Seslar SP Surgical approaches to epicardial pacemaker placement: does pocket location affect lead survival? Pediatr Cardiol 2010 9 7 1016 1024 10.1007/s00246-010-9754-1 20690018 PMC2948166

[B7] Silvetti MS Twenty years of paediatric cardiac pacing: 515 pacemakers and 480 leads implanted in 292 patients Europace 2006 9 7 530 536 10.1093/europace/eul062 16798767

[B8] Post MC Budts W Bruaene A Willems R Meyns B Rega F Gewillig M Failure of epicardial pacing leads in congenital heart disease: not uncommon and difficult to predict Neth Heart J 2011 9 7–8 331 335 21567217 10.1007/s12471-011-0158-5 PMC3144327

[B9] Murayama H Maeda M Sakurai H Usui A Ueda Y Predictors affecting durability of epicardial pacemaker leads in pediatric patients J Thorac Cardiovasc Surg 2008 9 2 361 366 10.1016/j.jtcvs.2007.09.002 18242269

[B10] Odim J Suckow B Saedi B Laks H Shannon K Equivalent performance of epicardial versus endocardial permanent pacing in children: a single institution and manufacturer experience Ann Thorac Surg 2008 9 4 1412 1416 10.1016/j.athoracsur.2007.12.075 18355537

[B11] Fortescue EB Berul CI Cecchin F Walsh EP Triedman JK Alexander ME Comparison of modern steroid-eluting epicardial and thin transvenous pacemaker leads in pediatric and congenital heart disease patients J Interv Card Electrophysiol 2005 9 1 27 36 10.1007/s10840-005-3797-x 16311936

[B12] Batra AS Balaji S Pacing in adults with congenital heart disease Expert Rev Cardiovasc Ther 2006 9 5 663 670 10.1586/14779072.4.5.663 17081088

[B13] Kwak JG Kim SJ Song JY Choi EY Lee SY Shim WS Lee CH Lee C Park CS Permanent epicardial pacing in pediatric patients: 12-year experience at a single center Ann Thorac Surg 2012 9 2 634 639 10.1016/j.athoracsur.2011.09.072 22192754

[B14] Kubus P Materna O Gebauer RA Matejka T Gebauer R Tlaskal T Janousek J Permanent epicardial pacing in children: long-term results and factors modifying outcome Europace 2012 9 4 509 514 10.1093/europace/eur327 21993433

[B15] Janousek J van Geldorp IE Krupickova S Rosenthal E Nugent K Tomaske M Früh A Elders J Hiippala A Kerst G Gebauer RA Kubus P Frias P Gabbarini F Clur SA Nabel B Ganame J Papagiannis J Marek J Tisma-Dupanovic S Tsao S Nürnberg JH Wren C Friedberg M de Guillebon M Volaufova J Prinzen FW Delhaas T Working Group for Cardiac Dysrhythmias and Electrophysiology of the Association for Pediatric Cardiology Permanent cardiac pacing in children: choosing the optimal pacing site: a multicenter study Circulation 2013 9 5 613 623 10.1161/CIRCULATIONAHA.112.115428 23275383

